# Maternal Diglyceride Diet Attenuates Selected Metabolic Effects of Maternal High‐Fat Feeding in Offspring via Energy Metabolism and Microbiota Modulation

**DOI:** 10.1002/fsn3.72427

**Published:** 2026-10-13

**Authors:** Qiu Wu, Chun Li, Shihao Song, Hanyue Wang, Rentang Zhao, Luyan Sun, Bingqing Shang, Yue Geng

**Affiliations:** ^1^ Key Laboratory of Food Nutrition and Safety of SDNU, Provincial Key Laboratory of Animal Resistant Biology, College of Life Science Shandong Normal University Jinan People's Republic of China

**Keywords:** diglyceride, gut microbiota‐carnitine, mother mice, obesity, offspring

## Abstract

Diglyceride (DAG) has been shown to improve obesity in animals, but its effects on mothers and offspring were unclear. This study investigated whether maternal DAG intake affects male offspring by mating female C57BL/6J mice on a DAG diet with normal males. Male offspring were weaned at 3 weeks, with some euthanized and others fed normal, high‐fat (HFD), or DAG diets for 9 weeks. Results showed that offspring from DAG‐fed mothers had lower blood lipids than those from HFD‐fed mothers, and maternal DAG intake was associated with restoration of purine/pyrimidine metabolism while altering gut microbiota (lower *Firmicutes*/*Bacteroidetes* ratio, higher butyrate). Fecal transplants confirmed DAG partially reversed HFD‐induced gut dysbiosis. Although DAG mitigated maternal obesity effects, post‐weaning diet had a stronger influence, suggesting DAG oil may help prevent intergenerational obesity transmission.

## Graphical abstract

Maternal consumption of diacylglycerol (DAG) oil attenuates high‐fat diet (HFD)‐induced obesity and dyslipidemia in mother mice and their male offspring. DAG restored purine/pyrimidine metabolism, maintained mitochondrial morphology, suppressed the DAG‐PKCβ/PKD1‐NF‐κB pathway, and promoted fatty acid oxidation (CPT1A↑, ACSS2↓). It also modulated the gut microbiota (lower Firmicutes/Bacteroidetes ratio, elevated butyrate), and fecal microbiota transplantation confirmed that DAG partially reversed HFD‐induced gut dysbiosis. Although DAG mitigated the effects of maternal obesity, the post‐weaning diet exerted a stronger influence, suggesting that DAG oil may help prevent intergenerational obesity transmission.

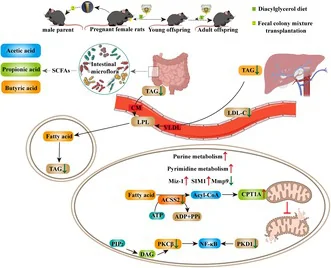

## 1 Introduction

The incidence of global obesity is on the rise. In 2025, the global obese population had exceeded 1 billion, accounting for about one‐eighth of the total global population. Among them, about 159 million obese people are children or adolescents aged 5 to 19, and 879 million are adults. Obesity poses a huge threat to the health of both adults and children (NCD Risk Factor Collaboration (NCD‐RisC) 2024). The nutritional and physiological status of the mother is an important determinant of the health of the offspring (Sun et al. 2020). Maternal obesity caused by diet can lead to an increased susceptibility of obesity in the offspring (Chang et al. 2019). Changes in body fat distribution (Kentistou et al. 2023), intestinal flora (Devi et al. 2023) and serum metabolism (Greenhill 2017) brought by obesity itself may also have a lasting impact on the long‐term health of the offspring through the bond of the mother. However, the manifestation of maternal effects in offspring is different in different genders, with male offspring exhibiting more severe metabolic syndrome‐like phenotypes than female offspring (Chang et al. 2019; Wankhade et al. 2018). Therefore, the health status of pregnant women and the uterus environment will have long‐term effects on the lives of newborns and offspring in adulthood. The impact of maternal obesity on offspring includes intrauterine nutritional status and metabolic changes, epigenetic modifications, etc.

Previous studies have shown that diacylglycerols (DAG), especially 1,3‐DAG, have certain beneficial effects on metabolic syndrome such as obesity. DAG has been found to inhibit intra‐abdominal fat accumulation (Murase et al. 2001), reduce postprandial serum TAG levels (Xu et al. 2018) and cholesterol levels (Prabhavathi Devi et al. 2018), improve glucose metabolism (Choi et al. 2015; Shoji et al. 2012), reduce thrombosis (Ijiri et al. 2006) and atherosclerosis risks (Fujii et al. 2007). A diet rich in DAG can reduce weight gain, visceral fat weight (Murase et al. 2001) and visceral fat area (Saito et al. 2016) in obese mice, and it can also improve insulin resistance induced by high‐fat diet by inhibiting hepatic gluconeogenesis and stimulating fat oxidation in skeletal muscles (Saito et al. 2007). These results provide evidence for the protective effect of DAG in obesity. However, it is currently unclear whether the protective effects of DAG on obesity extend to offspring and the potential cross‐effects of maternal diet and post‐weaning diet on offspring obesity.

The gut microbiota plays a critical role in the development of obesity. The dysbiosis of obesity is usually characterized by a reduction of microbial diversity (Le Chatelier et al. 2013), changes in the relative abundances of major phyla (such as *Firmicutes* and *Bacteroidetes*), and/or the overgrowth of pathogenic microorganisms (Murphy et al. 2010). Studies have shown that germ‐free (GF) mice cannot gain weight when treated with high‐fat or high‐fructose diet (Bäckhed et al. 2007), however, when adult GF mice received gut microbiota from normal mice, they rapidly gain weight and are insulin resistant (Bäckhed et al. 2004). GF mice receiving fecal colony mixture transplantation (FCMT) from obese twin donors exhibit significantly increased fat mass compared to those receiving FCMT from lean twin donors (Ridaura et al. 2013). Other research suggested that diacylglycerols (DAG) can lead to significant changes in the gut microbiota in mice (Han et al. 2022). Maternal microbial factors can be altered by maternal obesity, affecting the establishment and composition of the initial microbiota in offspring, as well as subsequent development and metabolic regulation, thereby influencing future health and disease (Magalhães et al. 2024; Miyamoto et al. 2024). Metabolites produced by gut bacteria, such as SCFAs, are key agents of host‐microbial reciprocity, thereby affecting host lipid metabolism and energy homeostasis (Wang, De Paepe, et al. 2023). However, the potential impact of DAG‐induced changes in maternal gut microbiota and SCFAs on the development of obesity and metabolic disorders in adult offspring is not yet clear.

This study aims to investigate the potential mechanisms by which maternal factors, through DAG dietary intervention in mice, may influence offspring obesity by examining the gut microbiota and SCFAs levels. It also seeks to observe the effects of DAG on gut microbiota dysbiosis induced by a high‐fat diet and determine if FCMT can reproduce the effects of DAG in mice on a normal diet. This research will provide a better understanding of the complex interactions between maternal metabolic changes and alterations in gut microbiota composition induced by DAG, and the impact of offspring exposure to a high‐fat diet on the development of obesity. It may offer potential insights for future preclinical research in rodent models on metabolic disorders and obesity and provide theoretical support for further studies investigating diglyceride edible oils as potential healthy fats in appropriate animal models, though translation to humans requires caution due to species differences in lipid metabolism and the presence of cecotrophy in rodents.

## 2 Materials and Methods

### 2.1 Mice and Diets

Female C57BL/6J mice were purchased from Beijing Vital River Laboratory Animal Technology Co. Ltd. Mice were randomized into experimental groups using a computer‐generated random sequence. To minimize bias, cage positions were randomized, and investigators collecting samples remained blinded to the group identities. The whole experimental design diagram was shown in Figure 1. These animals were randomly divided into three groups: (1) normal diet (mND) group, fed a standard diet (10 kcal% fat); (2) high‐fat diet (mHFD) group (60 kcal% fat, D12492, research diet); (3) diacylglycerol (DAG) diet (mDAG) group. The oil content of DAG feed was 34.9%, and the oil in the high‐fat diet was replaced by 40% diacylglycerol oil from Guangdong Shanbainian Special Medical Food Co. Ltd. The feed in this experiment was processed by Ready Dietech (Shenzhen) Co. Ltd. and sterilized by using irradiation. Ethical approval for the animal experiment in this study was granted by Shandong Normal University Research Ethics Committee, Reference number AEECSDNU2022125, dtd 10/26/2022. After 4 weeks of continuous feeding, female mice were mated with male mice fed a normal diet, and the diet grouping of pregnant and lactating female mice remained unchanged. 168 mice were born, 84 males and 84 females. On Day 21 after birth, all female mice and 10 young offspring in every group (3 weeks old) were euthanized under anesthesia; the serum, liver and adipose tissue samples from both the mother mice and young offspring, as well as feces from the mother mice, were collected for analysis.

**FIGURE 1:**
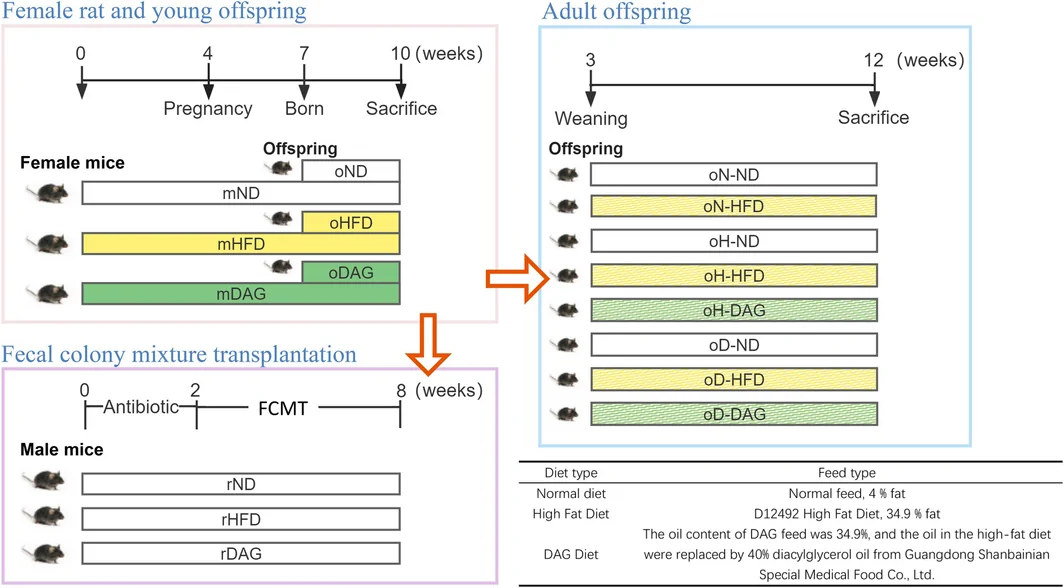
Animal experimental design.

Then the rest of the female offspring were used for the follow experiments. Offspring from the normal diet group were divided into two groups, oN‐ND group and oN‐HFD group (fed normal diet and high‐fat diet, respectively). Offspring from the mHFD group and mDAG group were divided into three groups, respectively. They were the oH‐ND group, oH‐HFD group, and oH‐DAG group (fed normal diet, high‐fat diet, and DAG diet, respectively) and the oD‐ND group, oD‐HFD group, and oD‐DAG group (fed normal diet, high‐fat diet, and DAG diet, respectively). After 9 weeks of continuous feeding, all adult offspring (12 weeks old) were euthanized under anesthesia, and the serum, liver, adipose tissue, and feces were collected for analysis. Both young and adult offspring used in this experiment were males.

The collected fresh feces from the same experimental group of mice were mixed. 500 mg of fresh feces were suspended in 5 mL of PBS buffer containing 0.5 g/L of cysteine as a reducing agent. After homogenizing and vortex mixing, the suspension was centrifuged at 440 *g for 3 min. The supernatant was collected as the FCMT solution. In the first week, the mice were orally gavaged once a day for three consecutive days (200 μL per mouse). In the remaining weeks, the fecal suspension was introduced every other day to enhance colonization. Throughout the experiment, all mice maintained a normal diet, and their body weight and food intake were dynamically monitored. After 5 weeks of continuous fecal transplantation, the mice were given a 1‐week buffer period. At the end of the experiment, all mice were euthanized under anesthesia, and fat tissue, liver, and feces were collected for analysis.

For multi‐omics, histological, and molecular analyses, three animals were randomly selected per group from the available biological replicates (10 dams/young offspring per group; six adult offspring per group) using a random number generator. These *n* = 3 represent independent biological replicates (distinct individuals), not technical replicates. Where multiple tissue‐based assays were performed (H&E, electron microscopy, RT‐qPCR), tissues were harvested from the same three animals to enable integrated interpretation. Fecal samples for SCFA and 16S rRNA analyses, as well as serum for 1H‐NMR, were similarly collected from these same selected animals.

### 2.2 Serum Biochemical Index Detection

The blood samples were centrifuged in 1650*g for 15 min, the supernatant was serum samples and they were collected. Reagent kits were used to measure the levels of triglycerides (TG), total cholesterol (T‐CHO), low‐density lipoprotein cholesterol (LDL‐C), and high‐density lipoprotein cholesterol (HDL‐C).

### 2.3 Tissue Pathological Examination

Liver tissues were fixed in 4% paraformaldehyde for 24 h, dehydrated, embedded in paraffin, and then 5 μm tissue sections were prepared. The sections were then stained with hematoxylin and eosin (H&E), then observed and photographed by using a Nikon Eclipse E100 microscope (Nikon, TKY, Japan). Histological assessment of lipid accumulation and morphological alterations was performed by two independent pathologists in a blinded manner using a standardized scoring system.

### 2.4 Electron Microscopic Observation

Liver tissues were fixed in Gluta fixative solution (for electron microscopy, 2.5%), embedded in paraffin after routine processing, and sliced into 60–70 nm sections. The sections were stained with uranyl acetate and lead citrate. The ultrastructure of liver cells was observed and photographed by using a Hitachi Corporation (Beijing, China) HT‐7800 electron microscope.

### 2.5 Measurement of Gene Expression

Total RNA was extracted from mouse liver and adipose tissue. The expression levels of lipid metabolism related genes (Pparα, Cpt1a, and Acss2), DAG‐PKC signaling pathway related genes (Pkcβ and Pkd1), and obesity‐associated genes previously linked to differential methylation (Miz‐1, Sim1, and Mmp9) were detected by quantitative reverse transcription and polymerase chain reaction (RT‐qPCR). GAPDH was used as the reference gene, and the results were processed using the ΔΔCT method, with the expression fold change calculated as 2^−ΔΔCT^. The primer sequences were shown in Table 1 and were synthesized by Wuhan Servicebio Biotechnology Co. Ltd. (Wuhan, China).

**TABLE 1:** Primer sequences.

Gene name	Forward (5′‐3′)	Reverse (5′‐3′)
*Gapdh*	CCTCGTCCCGTAGACAAAATG	TGAGGTCAATGAAGGGGTCGT
*Pparα*	CACTACGGAGTTCACGCATGT	GTGACATCCCGACAGACAGGC
*Acss2*	GTTCTGCTTCTTTCCCATTCTTC	GCTGCTTGAACACCAGATAACC
*Cpt‐1a*	GCACGAGGAAAAAATAAGCAATCT	TTGTCAAACCACCTGTCGAAAC
*Pkcβ*	GGGATGAAATGCGACACCTGTA	GGATTGAGGGAACACTTGATGGT
*Pkd1*	CTAGACCTGTCCCACAACCTACTT	AGCAAACACGCCTTCTTCTAATGT
*Miz‐1*	AGACTCACGACACCGACAAGGA	TCCCTGAGGTGGTGAACTGCT
*Mmp9*	CTCGGGAAGGCTCTGCTGTT	AACTCACACGCCAGAAGAATTTG
*Sim1*	CCACTTACTGTTGGTGAAGGGAC	GTGAGGACGTAGTTGACGCTGA

Each RT‐qPCR reaction was performed in technical triplicate for each biological sample, but the reported *n = 3* refers to biologically independent animals, not technical replicates.

### 2.6 1H‐NMR Analysis

The mouse serum samples were analyzed using a Bruker AVANCE III 400 nuclear magnetic resonance spectrometer (Bruker AVANCE III 400, Karlsruhe, Germany) for 1H‐NMR spectrum collection. The spectra were processed using MestReNova software, and multivariate statistical analysis of the data was performed using SIMCA 14.1 software (UMetrics AB, Umea, Sweden). Potential biomarkers were selected based on VIP values and they were compared with the Human Metabolome Database (HMDB). Relevant biochemical reactions were discovered and identified through the Kyoto Encyclopedia of Genes and Genomes (KEGG) and HMDB databases.

### 2.7 Short‐Chain Fatty Acid Analysis

The content of short‐chain fatty acids (SCFAs) in feces was determined using a Thermo Trace 1300 gas chromatography system and Thermo ISQ 7000 mass spectrometer (Thermo Fisher Scientific, USA). An appropriate amount of fecal sample was placed in a 2 mL centrifuge tube, added with 50 μL of 15% phosphoric acid, 100 μL of internal standard solution (4‐methylvaleric acid) at a concentration of 125 g/mL, and 400 μL of ether, followed by mixing for 1 min. The mixture was then centrifuged at 12,000 rpm at 4°C for 10 min, and the supernatant was taken for analysis. The chromatographic conditions were as follows: Agilent HP‐INNOWAX capillary column (30 m × 0.25 mm ID × 0.25 μm); split injection with an injection volume of 1 μL and a split ratio of 10:1. The injection port temperature was set at 250°C, ion source temperature at 300°C, and transfer line temperature at 250°C. The temperature program started at 90°C, ramped up to 120°C at a rate of 10°C/min, further increased to 150°C at 5°C/min, and finally raised to 250°C at 25°C/min and held for 2 min. Helium was used as the carrier gas with a flow rate of 1.0 mL/min. Mass spectrometry conditions included electron ionization (EI) source, selected ion monitoring (SIM) scan mode, and electron energy set at 70 eV.

### 2.8 Microbial Flora Analysis

The feces of mice collected from microflora were stored at −80°C. 16S rRNA sequencing of fecal microorganisms was commissioned by Novogene Co. Ltd. (Beijing, China). The final ASVs are obtained by denoising the valid data through DADA2 and filtering out the sequences whose abundance is less than 5. The representative sequences of each ASV were annotated to obtain corresponding species information and species‐based abundance distribution.

### 2.9 Statistical Analysis

All data are presented as Mean ± SEM. Statistical analysis was performed using IBM SPSS Statistics 26 and GraphPad Prism 7 software. Normality and homogeneity of variance were verified prior to analysis. Group comparisons were conducted using independent samples *t*‐tests for two groups and one‐way analysis of variance (ANOVA) for multiple groups. Post hoc comparisons were performed using Tukey's HSD tests. Spearman correlation analysis was used to calculate correlation coefficients. A significance level of *p* < 0.05 was considered statistically significant, and *p* < 0.01 was considered highly significant.

## 3 Results

### 3.1 Dietary Diglycerides Improved Lipid Abnormalities in Both Mother Mice and Their Offspring at a Young Age and Adult Age (12 Weeks Old)

The data on lipid changes were shown in Table 2. Compared to obese mother mice induced by HFD, the mother mice fed with DAG diet showed reduced body weight gain, decreased white adipose tissue weight, and lowered serum TG and T‐CHO levels, suggesting a potential protective effect of the DAG diet on mother mice. However, compared with the normal diet (ND) group, HDL‐C levels were significantly decreased in HFD‐fed dams, and DAG intervention failed to restore them. At weaning, offspring of HFD‐fed dams exhibited higher body weight gain and a greater proportion of white adipose tissue weight compared with offspring of ND‐fed dams. Similar to the results observed in dams, offspring of DAG‐fed dams showed decreased serum TG, T‐CHO, and LDL‐C levels, while their HDL‐C levels remained significantly lower than those of the ND group. These preliminary results suggest that the anti‐obesity effects of DAG can be transmitted to offspring through breastfeeding.

**TABLE 2:** The effect of diglycerides on mother mice, offspring and progeny of cecal transplants.

	Body weight gain (g)	Body weight growth rate (%)	iWAT weight/body weight (%)	eWAT weight/body weight (%)	TG (mmol/mL)	T‐CHO (mmol/mL)	LDL‐C (mmol/mL)	HDL‐C (mmol/mL)
Female mice (*n* = 10)								
mND	11.86 ± 0.62	83.57 ± 4.46	1.53 ± 0.56	0.43 ± 0.07	0.63 ± 0.01	3.31 ± 0.18	1.32 ± 0.08	3.54 ± 0.13
mHFD	14.81 ± 0.99*	110.07 ± 8.13**	1.51 ± 0.15	1.98 ± 0.33**	0.71 ± 0.01**	3.75 ± 0.31	1.57 ± 0.29	2.29 ± 0.20*
mDAG	10.08 ± 0.66^##^	75.08 ± 4.38^##^	1.08 ± 0.12	1.02 ± 0.21^#^	0.51 ± 0.02**^##^	2.67 ± 0.11^##^	1.28 ± 0.11	2.19 ± 0.10**^##^
Young offspring (3 weeks, *n* = 10)								
oND	3.34 ± 0.26	50.53 ± 3.35	0.425 ± 0.032	0.211 ± 0.028	0.91 ± 0.06	2.85 ± 0.15	1.06 ± 0.10	13.07 ± 1.00
oHFD	6.25 ± 0.20**	91.27 ± 3.27**	0.562 ± 0.026*	0.514 ± 0.055**	1.48 ± 0.04**	4.10 ± 0.10**	1.81 ± 0.10**	6.69 ± 0.34**
oDAG	5.70 ± 0.19**	88.56 ± 3.07**	0.534 ± 0.052	0.394 ± 0.062*	0.46 ± 0.02**^##^	2.32 ± 0.25**^##^	1.26 ± 0.12^##^	6.35 ± 0.28**
Adult offspring (12 weeks, *n* = 6)								
oN‐ND	13.34 ± 0.87^b^	90.67 ± 12.99^b^	0.33 ± 0.02^d^	0.61 ± 0.05^d^	0.60 ± 0.04^b^	1.89 ± 0.06^de^	0.80 ± 0.34^b^	3.31 ± 0.20^c^
oN‐HFD	17.75 ± 0.96^a^	112.01 ± 16.44^ab^	1.05 ± 0.34^bc^	2.15 ± 0.62^bc^	0.79 ± 0.10^ab^	4.55 ± 0.53^ab^	2.99 ± 0.60^a^	3.93 ± 0.17^bc^
oH‐ND	13.55 ± 1.09^b^	104.37 ± 18.49^ab^	0.41 ± 0.03^d^	0.65 ± 0.07^d^	0.66 ± 0.09^b^	2.53 ± 0.09^de^	1.19 ± 0.44^b^	3.36 ± 0.43^c^
oH‐HFD	19.49 ± 0.92^a^	128.66 ± 6.94^a^	1.76 ± 0.24^a^	3.68 ± 0.65^a^	0.89 ± 0.03^a^	4.98 ± 0.28^a^	2.78 ± 0.90^ab^	4.17 ± 0.29^ab^
oH‐DAG	13.89 ± 0.85^b^	116.65 ± 6.92^ab^	0.62 ± 0.11^cd^	1.55 ± 0.31^cd^	0.85 ± 0.05^ab^	3.13 ± 0.39^cd^	1.24 ± 0.52^b^	4.78 ± 0.37^a^
oD‐ND	12.84 ± 0.76^b^	99.51 ± 7.91^ab^	0.41 ± 0.02^d^	0.61 ± 0.07^d^	0.62 ± 0.04^b^	1.75 ± 0.14^e^	0.89 ± 0.10^b^	2.52 ± 0.16^d^
oD‐HFD	17.59 ± 1.02^a^	118.13 ± 10.72^ab^	1.48 ± 0.23^ab^	3.05 ± 0.55^ab^	0.78 ± 0.08^ab^	3.79 ± 0.42^bc^	2.09 ± 0.72^ab^	3.82 ± 0.20^bc^
oD‐DAG	13.10 ± 0.61^b^	107.93 ± 4.24^ab^	0.61 ± 0.08^cd^	1.17 ± 0.12^cd^	0.68 ± 0.08^ab^	2.90 ± 0.30^cde^	1.11 ± 0.24^b^	4.11 ± 0.28^ab^
Fecal colony mixture transplantation (*n* = 6)								
rND	1.14 ± 0.02	2.16 ± 0.04	0.388 ± 0.05	0.706 ± 0.12	/	/	/	/
rHFD	0.89 ± 0.34	1.68 ± 0.65	0.32 ± 0.04	0.681 ± 0.08	/	/	/	/
rDAG	1.48 ± 0.19	2.78 ± 0.36	0.46 ± 0.04	0.573 ± 0.10	/	/	/	/

*Note:* iWAT: Inguinal white fatty tissue; eWAT: Epigonadal white adipose tissue. For maternal and young offspring groups: *p < 0.05, **p < 0.01 vs. mND or oND, ^#^ *p* < 0.05, ^##^ *p* < 0.01 vs. mHFD or oHFD. For adult offspring groups, values with different superscript letters (a–e) are significantly different (*p* < 0.05, Tukey's HSD test). Data are mean ± SEM.

The health status of the mother has long‐term effects on the offspring, so relevant analyses were conducted on the adult offspring (12 weeks old). As shown in Table 2, when faced with the challenge of a high‐fat diet after weaning, the offspring of high‐fat diet mothers (oH‐HFD) had higher weight gain than the offspring of normal diet mothers (oN‐HFD), and the offspring of DAG‐diet mothers (oD‐HFD) showed lower adipose tissue weight than the offspring of high‐fat diet mothers (oH‐HFD). Compared with the adult offspring with normal diet (oN‐ND), the normal diet (oH‐ND, oD‐ND) after weaning helped to restore the serum TG, T‐CHO, LDL‐C levels of the adult offspring, although the mother mice received a high‐fat diet and a DAG diet, respectively. Adult offspring on a post‐weaning DAG diet (oH‐DAG) had lower serum TG, T‐CHO, and LDL‐C levels than adult offspring on a post‐weaning high‐fat diet (oH‐HFD). The adult progenies (oD‐ND, oD‐HFD, oD‐DAG) of DAG‐diet mothers showed lower serum TG, T‐CHO, LDL‐C levels than those of high‐fat diet mothers (oH‐ND, oH‐HFD, oH‐DAG). Unlike female mice and young male offspring, LDL‐C levels were increased in male adult offspring.

### 3.2 The DAG Diet Maintains Mitochondrial Morphology in Maternal Mice and Promotes the Expression of Genes Related to Lipid Metabolism in Juvenile Offspring

Obesity can lead to abnormal lipid deposition, mitochondrial structural abnormalities, and lipid metabolism disorders. Liver histology showed that compared to the ND group, mother mice receiving HFD had more and larger lipid droplets in the liver (Figure 2a, black arrows); in contrast, mother mice receiving DAG diet had reduced lipid droplets compared to those receiving HFD (Figure 2a, black arrows). Electron microscopy images revealed that the liver cells of HFD‐fed mother mice exhibited altered mitochondrial morphology, with blurred membrane structures and incomplete cristae (yellow arrows), which were partially improved in the DAG group of mother mice (Figure 2b). Offspring of HFD‐fed mother mice (oHFD) showed more pronounced hepatic lipid accumulation (Figure 2f, black arrows) and mitochondrial structural damage (Figure 2g, yellow arrows) compared to the other two groups. In comparison to high‐fat diet, DAG diet appeared to improve the expression of lipid metabolism‐related genes (Ppar, Acss2, Cpt1a) in mother mice and their young offspring (Figure 2c–e,h–j). DAG diet in female mice can reduce lipid deposition, maintain normal mitochondrial morphology, promote the expression of genes related to lipid metabolism, and thus increase the resistance of offspring to the adverse effects of maternal HFD. To further investigate the effects of DAG diet on maternal metabolism and epigenetics, a series of experiments were conducted on maternal serum, as well as liver and adipose tissue of mother mice and their young offspring.

**FIGURE 2:**
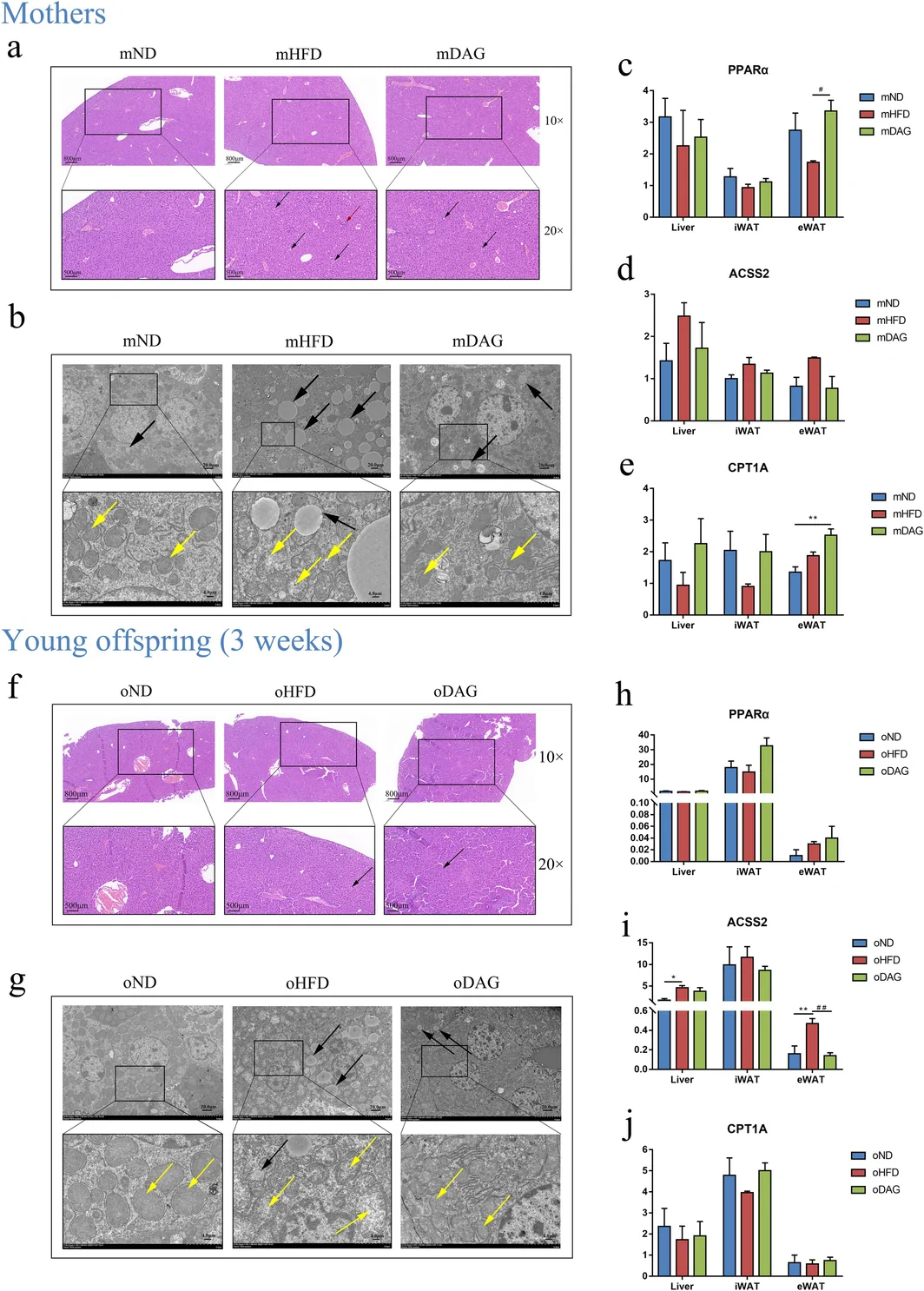
The effects of diglyceride on lipid accumulation in mother mice and their offsprings (*n* = 3). (a) H&E stained liver sections of mother mice; (b) TEM observation of liver sections of mother mice; (c–e) Expression levels of lipid metabolism‐related genes in the liver and adipose tissue of mother mice; (f) H&E stained liver sections of young offspring; (g) TEM observation of liver sections of young offspring; (h–j) Expression levels of lipid metabolism‐related genes in the liver and adipose tissue of young offspring. *p < 0.05, **p < 0.01 vs. mND or oND; #*p* < 0.05, ##*p* < 0.01 vs. mHFD or oHFD.

### 3.3 The DAG Diet Improves Maternal Serum Metabolism and Maintains Epigenetic Stability of Obesity‐Associated Genes

In order to determine the effects of DAG diet on serum and differential methylation gene expression, 1H‐NMR analysis and differential methylation gene (Miz‐1, SIM1, Mmp9) analysis were performed on serum, liver, and adipose tissue of mother and young offspring, respectively.

Orthogonal partial least squares discriminant analysis (OPLS‐DA) results showed good separation in the three groups of maternal serum (Figure 3a), and the confidence interval is good (Figure 3b). Based on the screening with VIP > 1.5, compared to the mND group, 69 differential metabolites were identified in the mHFD group, with 19 showing an upward trend and 50 showing a downward trend (Table S1), and pathway analysis was conducted on the differential metabolites (Figure 3c), involving 13 metabolic pathways, mainly focused on purine metabolism, pyrimidine metabolism, and interconversion of pentose and glucuronate. Compared to the mHFD group, 95 differential metabolites were identified in the mDAG group, with 84 showing an upward trend and 11 showing a downward trend (Table S2), and pathway analysis was conducted on the differential metabolites (Figure 3d), involving 19 metabolic pathways, mainly focused on purine metabolism, pyrimidine metabolism, and biosynthesis of valine, leucine, and isoleucine. Compared to the mND group, more differential metabolites in the purine metabolism and pyrimidine metabolism pathways in the mHFD group showed a downward trend; while compared to the mHFD group, more differential metabolites showed an upward trend in the mDAG group (Figure 3e).

**FIGURE 3:**
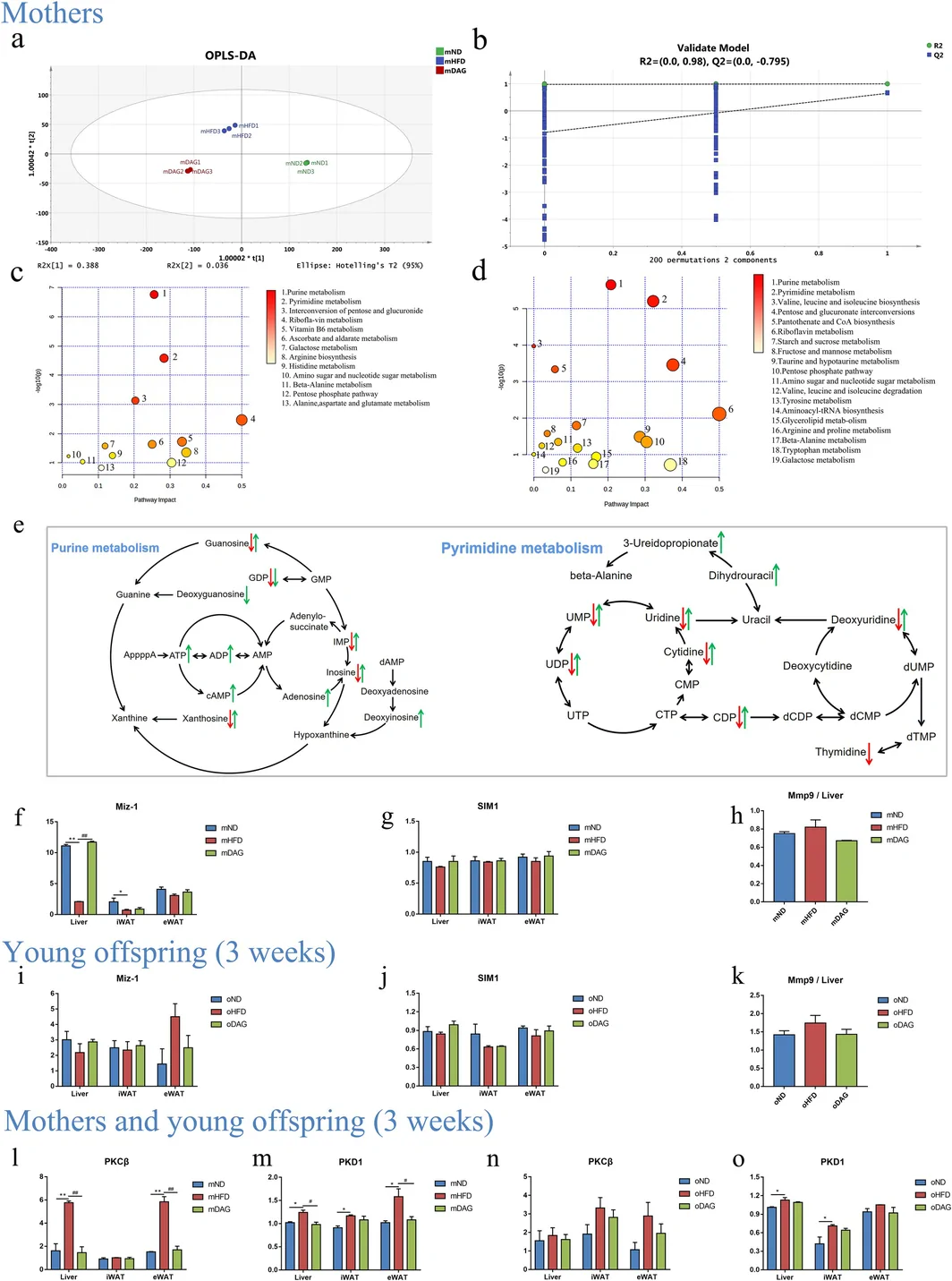
The effects of diglyceride on serum of mother mice and gene expression in mother mice and young offspring (*n* = 3). (a) Orthogonal Partial Least Squares Discriminant Analysis (OPLS‐DA); (b) Confidence test; (c) Differential metabolite pathway analysis between mND group and mHFD group; (d) Differential metabolite pathway analysis between mHFD group and mDAG group; (e) Changes in differential metabolites in the purine metabolism and pyrimidine metabolism pathways (red arrows indicate differential metabolite changes in the mHFD group compared to mND group, green arrows indicate differential metabolite changes in the mDAG group compared to mHFD group); Expression levels of obesity‐associated genes (Miz‐1, Sim1, and Mmp9) previously linked to differential methylation in the liver and white adipose tissue of mother mice (f–h) and young offspring (3 weeks old) (i–k). Expression levels of genes related to the DAG‐PKC signaling pathway in liver and white adipose tissue of mother mice (l, m) and young offspring (3 weeks old) (n, o) *p < 0.05, **p < 0.01 vs. mND; ^#^ *p* < 0.05, ^##^ *p* < 0.01 vs. mHFD.

Compared with high‐fat diet, the DAG diet was associated with improved stable expression of obesity‐associated genes previously implicated in differential methylation (Miz‐1, Sim1, Mmp9) (Figure 3f–h). Similar results were observed in young offspring (Figure 3i–k), while maintaining the stability of the DAG‐PKC signaling pathway in both maternal and young offspring (Figure 3l–o). These results suggested that the maternal DAG diet provides offspring with a healthy intrauterine environment and early developmental space by improving serum metabolism and maintaining gene expression homeostasis.

### 3.4 The DAG Diet Appeared to Improve the Gut Microbiota and Short‐Chain Fatty Acid Levels in Mothers and Their Adult Offspring (12 Weeks Old)

Sequencing analysis results showed that compared to the normal diet, the indices of α‐diversity (chao1, shannon) in the gut microbiota of high‐fat diet‐fed and DAG diet‐fed mothers decreased (Figure 4a,b). Compared to the high‐fat diet, the DAG diet appeared to improve the ratio of *Firmicutes* to *Bacteroidetes* in the gut microbiota of mothers (Figure 4c). Genus‐level species abundance clustering showed significant differences among mothers on a normal diet, high‐fat diet, and DAG diet, with the DAG diet improving the abnormal decrease in relative abundance of *Akkermansia* and *Alloprevotella* caused by the high‐fat diet, as well as the abnormal increase in relative abundance of *Helicobacter* and *Dubosiella* (Figure 4f).

**FIGURE 4:**
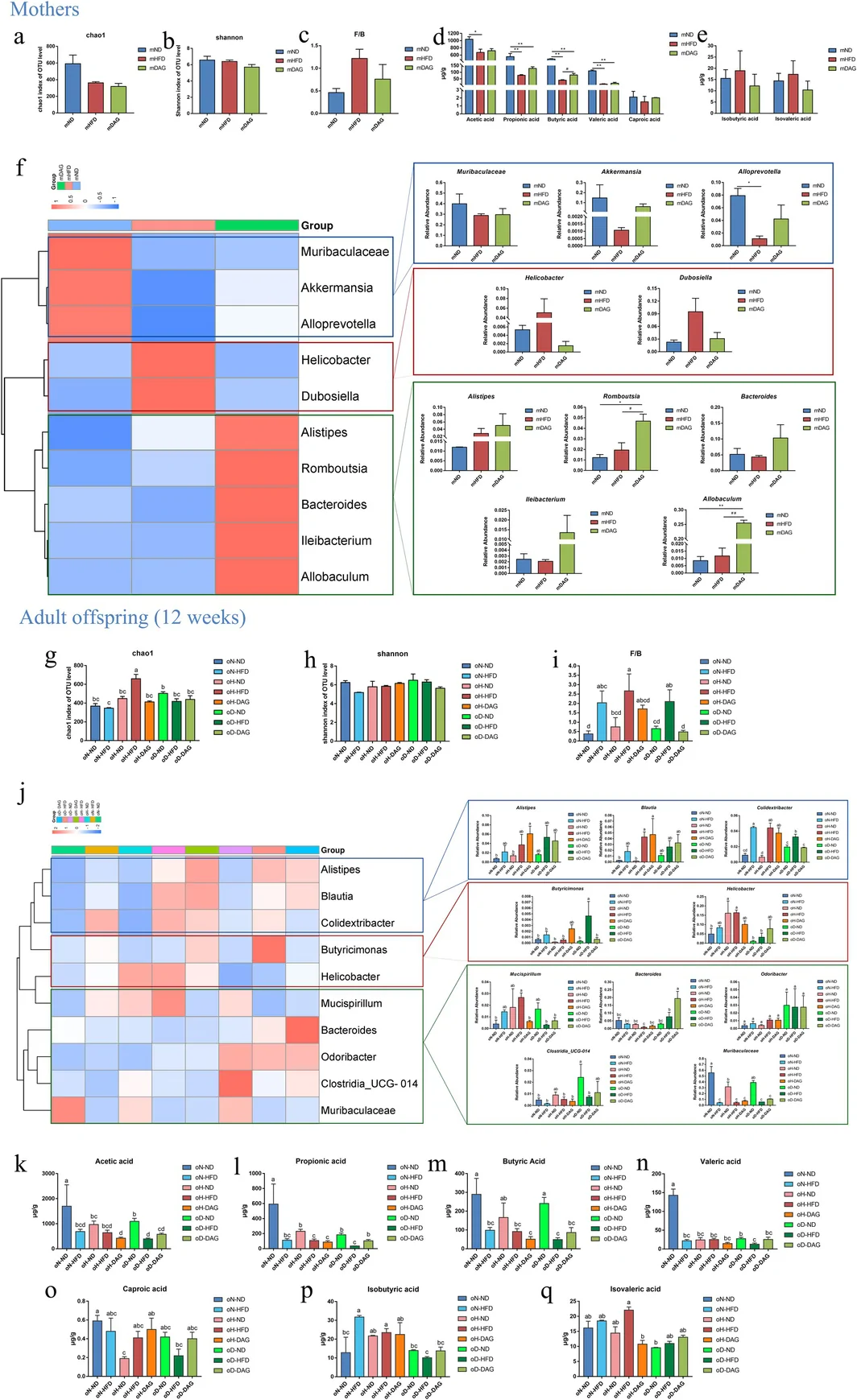
The effects of diglyceride on the gut microbiota and short‐chain fatty acid content in mother mice and adult offspring (12 weeks old) (*n* = 3). (a, b) Changes in α‐diversity indices (Chao1, Shannon) of gut microbiota in mother mice; (c) Ratio of *Firmicutes* to *Bacteroidetes* (F/B) in gut microbiota of mother mice; (d, e) Short‐chain fatty acid content in mother mice; (f) Cluster members and cluster features of different genera in the gut microbiota of mother mice, the bar graph shows the differences in the relative abundance of genera in different groups; (g, h) Changes in α‐diversity indices (Chao1, Shannon) of gut microbiota in adult offspring; (i) Ratio of *Firmicutes* to *Bacteroidetes* (F/B) in gut microbiota of adult offspring; (j) Cluster members and cluster features of different genera in the gut microbiota of adult offspring, the bar graph shows the differences in the relative abundance of genera in different groups; (k–q) Short‐chain fatty acid content in adult offspring. *p < 0.05, **p < 0.01 vs. mND; #*p* < 0.05, ##*p* < 0.01 vs. mHFD.

The DAG diet significantly improved the decrease in butyric acid caused by the high‐fat diet, and improved the decrease in acetic acid, propionic acid, valeric acid, and hexanoic acid levels, as well as the abnormal increase in isobutyric acid and isovaleric acid (Figure 4d,e). Interestingly, similar changes in α‐diversity were also observed in the adult offspring. Offspring of mothers on a high‐fat diet and continued high‐fat diet post‐weaning (oH‐HFD) showed the highest α‐diversity (Figure 4g,h). As shown in Figure 4i, post‐weaning high‐fat diet (oN‐HFD, oH‐HFD, oD‐HFD) led to an abnormal increase in the ratio of *Firmicutes* to *Bacteroidetes* in the gut microbiota of adult offspring. Comparatively, the DAG diet in mothers could improve this abnormality when compared to mothers on a high‐fat diet. Adult offspring of mothers on the DAG diet (oD‐ND, oD‐HFD, oD‐DAG) showed an overall lower ratio of *Firmicutes* to *Bacteroidetes* compared to adult offspring of mothers on a high‐fat diet (oH‐ND, oH‐HFD, oH‐DAG). Genus‐level species abundance clustering showed that post‐weaning normal diet could partially restore gut microbiota dysbiosis caused by mothers' high‐fat diet, and the adult offspring of mothers on the DAG diet exhibited higher resistance when facing a high‐fat diet (oD‐HFD) (Figure 4j). As shown in Figure 4k–q, post‐weaning normal diet partially restored the decrease in acetic acid, propionic acid, and butyric acid levels caused by mothers' high‐fat diet and DAG diet in adult offspring. Adult offspring of mothers on the DAG diet showed lower levels of isobutyric acid and isovaleric acid compared to offspring of mothers on the high‐fat diet.

### 3.5 Fecal Suspension Transplantation Changed Intestinal Flora Structure in Mice With Normal Diet

The gut microbiota is closely associated with obesity and related metabolic disorders. Therefore, FCMT was performed from mice with different dietary structures to mice on a normal diet. As shown in Table 2, there were no significant differences in weight and body fat among the groups. 16S rRNA gut microbiota analysis was conducted and the results showed that there were no significant differences in α‐diversity (Figure 5a,b) among the different groups, but the rHFD group receiving fecal transplantation from mice on a high‐fat diet had a higher ratio of *Firmicutes* to *Bacteroidetes* compared to other groups (Hildebrandt et al. 2009). Parabacteroides and *Akkermansia* are biomarkers for healthy people (Hu et al. 2022). The cluster heatmap results (Figure 5d) showed that the abundance of *Bacteroides* and *Helicobacter* increased in mice receiving fecal transplantation from mice on a high‐fat diet, while the abundance of *Akkermansia, Alloprevotella*, and *Parabacteroides* decreased. In contrast, the gut microbiota structure of mice receiving fecal transplantation from mice on a DAG diet (rDAG) was closer to that of mice on a normal diet, indicating that the DAG diet can indeed partially improve the gut microbiota dysbiosis induced by a high‐fat diet. Furthermore, it was found that gut microbiota transplantation could restore the *Firmicutes* to *Bacteroidetes* ratio and improve the abundance abnormalities at the genus level.

**FIGURE 5:**
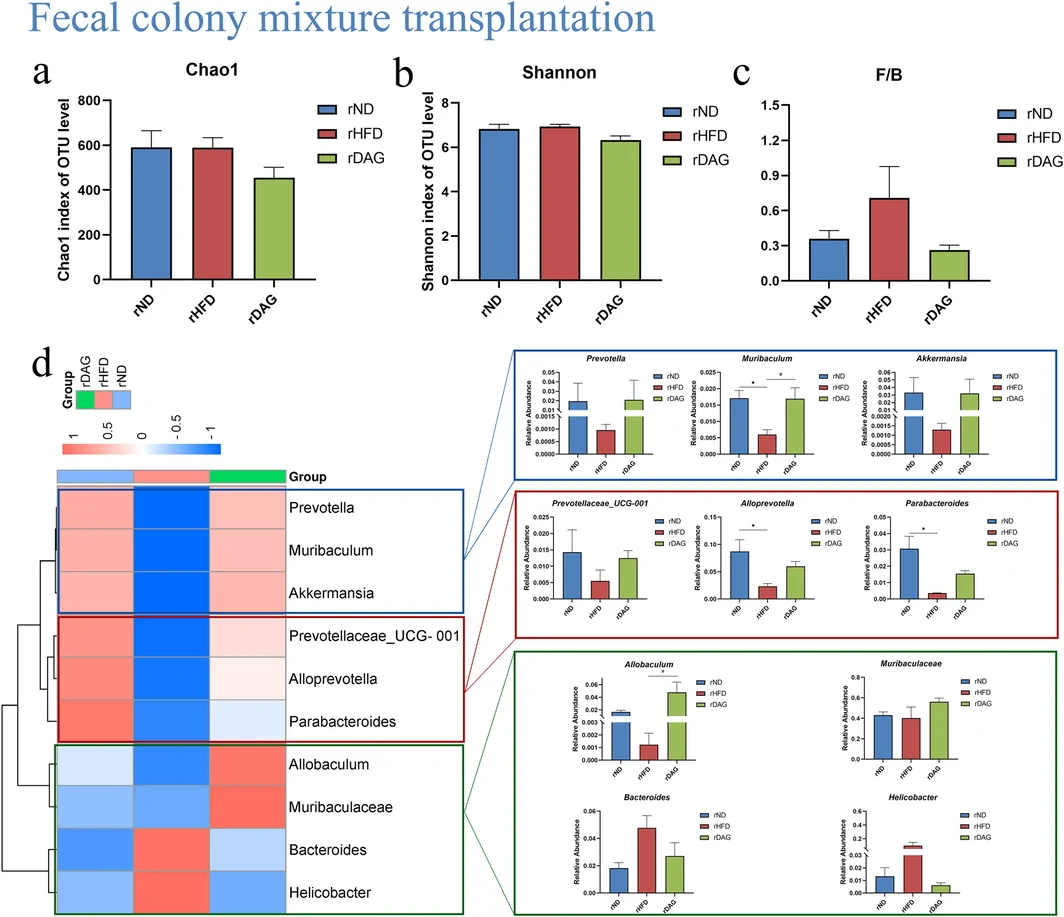
Effects of different dietary structure of fecal bacteria suspension transplantation on normal diet mice (*n* = 3). (a, b) Changes in α‐diversity indices (Chao1, Shannon) of gut microbiota in different groups of mice; (c) Ratio of *Firmicutes* to *Bacteroidetes* (F/B) in gut microbiota of different groups of mice; (d) Cluster members and cluster features of different gut microbiota genera. The bar graph shows the differences in the relative abundance of genera in different groups. #*p <* 0.05, vs. rDAG, *p < 0.05, vs. rHFD.

### 3.6 Correlation Between the Effects of DAG Diet on Mothers and Offspring

In order to understand the correlation of index changes between mother mice and their offspring, the index parameters of mother mice and their offspring were analyzed. The experimental results of phenotypic parameters and cytokine changes of female mice and their young male progeny (3 weeks old) were taken as horizontal and horizontal coordinates, Spearman correlation analysis was conducted, and the *p* value was selected to be 0.05, and the correlation heat map was drawn. As shown in Figure 6a, eWAT (%), the expression of PKCβ, PKD1, eWAT and ACSS2 in eWAT liver of female mice, the expression levels of PKCβ, PKD1 and ACSS2 in iWAT were correlated with the weight growth rate of young male offspring, the proportion of white adipose tissue, the expression levels of PKCβ in liver and adipose tissue, the expression levels of PKD1 in liver and iWAT, the expression levels of Miz‐1 in liver and eWAT, and the expression levels in liver and EWAT The expression level of Mmp9 was positively correlated. The expression level of Miz‐1 in liver and white adipose tissue, the expression of PPARα in liver and iWAT, and the expression of CPT1A in iWAT were negatively correlated with the growth rate of body weight and the percentage of white adipose tissue in body weight of young male offspring.

**FIGURE 6:**
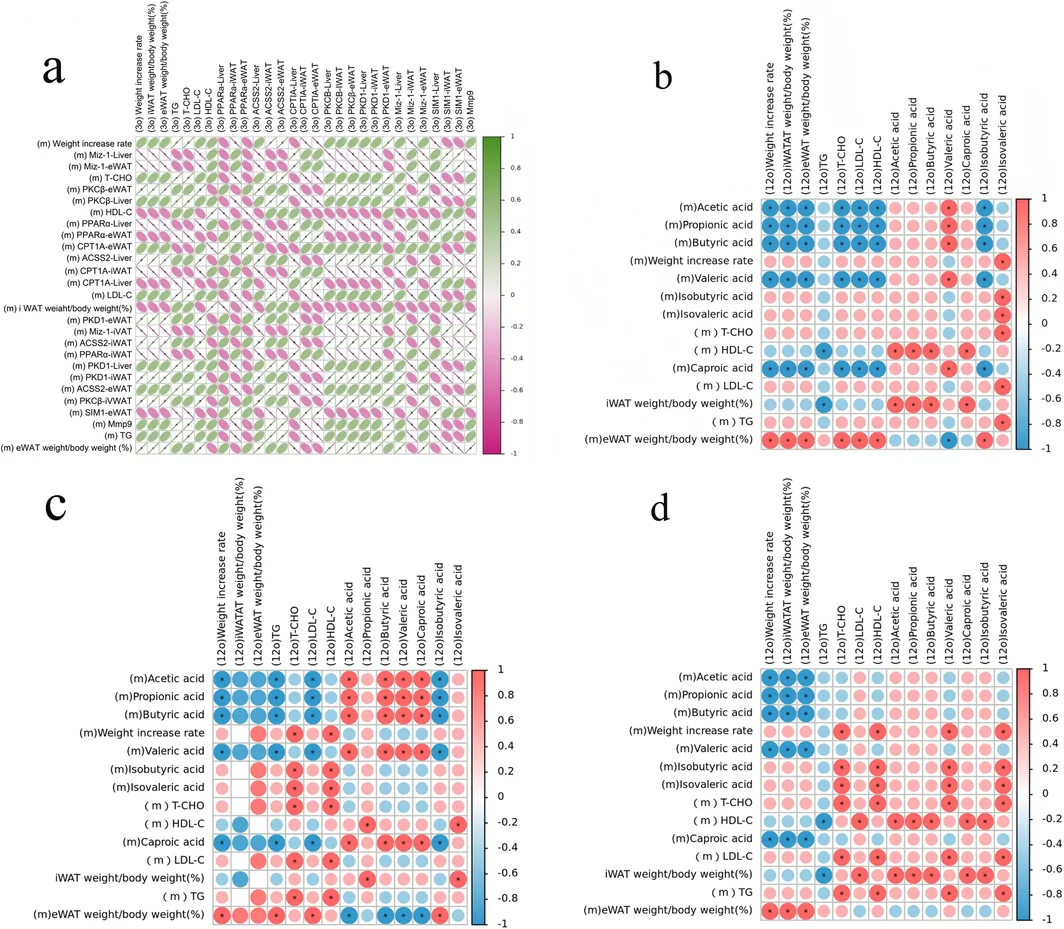
Correlation analysis of the effects of diglycerol on mothers and offspring (*n* = 3). (a) The correlation of phenotypic parameters and cytokines between female mice and young male offspring; (b) Adult male offspring maintained the same type of diet as their mothers, and their phenotypic parameters and SCFAs were correlated with those of their mothers.(c) Adult male offspring return to normal diet after weaning and their phenotypic parameters and SCFAs are correlated with those of mother mice; (d) Phenotypic parameters and SCFAs of adult male offspring who received a high‐fat diet after weaning were correlated with those of female mice.

The correlation analysis of phenotypic parameters and SCFAs between female mice and adult male progeny (12 weeks of age) was divided into three times. The first analysis included three groups of male progeny (oN‐ND group, oH‐HFD group, oD‐DAG group) whose dietary types were the same as those of female mice. The result was shown in Figure 6b. The changes of SCFAs content in female feces were negatively correlated with the growth rate of body weight, the proportion of white adipose tissue by weight, T‐CHO, and LDL‐C of male offspring in three constituent years. These results indicated that three different diet types can correlate the changes of SCFAs content in feces of female mice with the phenotypic indexes of adult offspring without any change in the diet type of adult offspring. The changes of maternal SCFAs metabolism level during the perinatal period can affect the obesity phenotype during the long‐term growth of adult offspring. The second analysis included three groups (oN‐ND group, oH‐ND group, oD‐ND group) in which both female and male offspring received a normal diet. The result was shown in Figure 6c. Although the three‐component offspring were influenced by three different diets from their mothers during infancy, they were all fed a normal diet after weaning. The correlations between the growth rate of body weight, the proportion of white adipocytes by weight, and SCFAs of their mothers were lower than those in oN‐ND group, oH‐HFD group, and oD‐DAG group. These results indicated that a healthy diet environment after weaning could help the adult offspring to gradually weaken the influence of maternal diet intervention and form a relatively independent growth trend in adult mice. The third analysis included three groups (oN‐HFD group, oH‐HFD group, oD‐HFD group) in which both female and male offspring received a high‐fat diet. The results were shown in Figure 6d. SCFAs in female feces were negatively correlated with the growth rate of body weight and the proportion of white adipose tissue in male offspring in three constituent years. This negative correlation suggested that DAG improves physical phenotype and metabolic parameters in female mice, and the protective effect of DAG in early life is more pronounced when adult offspring were exposed to a high‐fat diet after weaning.

## 4 Discussion

During the feeding period of maternal mice, the mHFD group showed a significantly higher increase in body weight compared to the mND group; compared to the mHFD group, the mDAG group exhibited a significant decrease in weight gain, with the mHFD group having the highest proportion of white adipose tissue weight. Prolonged high‐fat diet consumption led to abnormal changes in serum TG, T‐CHO, and LDL‐C levels in the mHFD group. In comparison to the mHFD group, the DAG diet significantly reduced TG and T‐CHO levels in maternal mouse serum while improving the elevated LDL‐C condition. It is worth noting that HDL‐C levels not only decreased in the mHFD group but also significantly decreased in the mDAG group. HDL‐C is generally considered a strong lipid predictor of cardiovascular disease (De Freitas et al. 2011; Emerging Risk Factors Collaboration et al. 2009). However, some research has yielded contradictory conclusions, suggesting a nonlinear relationship, akin to a U‐shaped trend (Ko et al. 2016; Madsen et al. 2017). Studies on stroke have indicated a nonlinear relationship between HDL‐C and stroke incidence, showing an inverse relationship when HDL‐C is below 1.55 mmol/L (Hu et al. 2023). Therefore, some researchers use the logarithm of TG/HDL‐C to represent the atherosclerosis index in plasma (AIP) (Amirkhizi et al. 2023; Wang, Qian, et al. 2023). For individuals with high non‐HDL‐C levels, lifestyle changes such as weight loss, reducing fat intake, substituting saturated fats with monounsaturated or polyunsaturated fats, increasing omega‐3 fatty acids, and soluble fiber intake can help reduce the risk of cardiovascular disease (Skretteberg et al. 2013; Byrne et al. 2017; Sacks et al. 2017). Therefore, the results of this experiment showed that the mDAG group of mother mice had decreased levels of TG and T‐CHO in serum; however, HDL‐C levels were not restored and remained significantly lower than those of both the ND and HFD groups. While these findings cannot directly resolve the ongoing debate on HDL‐C in humans, they provide preliminary data for comparative studies. During the feeding period of the offspring, the oND group of mice were healthy, active, without abnormal eating behavior or abnormal behaviors; the oHFD group and oDAG group of mice were respectively breastfed by mother mice fed with high‐fat diet and DAG diet, with a significant increase in body weight compared to the oND group; the oDAG group was lower than the oHFD group, but this decrease was not significant. The DAG diet can effectively reduce the levels of TG, T‐CHO, especially LDL‐C in mouse serum, indicating that mice fed with DAG diet have a reduced risk of atherosclerosis and improved blood lipid metabolism (Fernandez‐Friera et al. 2017). There is a strong correlation between the function of HDL in mothers and offspring (Stadler et al. 2023). Compared to fathers, maternal levels of T‐CHO, HDL‐C, and apoA1 are more strongly and consistently associated with postnatal weight in offspring, supporting a direct intrauterine effect (Øyri et al. 2023). Similar to the results in mother mice, the DAG diet also resulted in a decrease of HDL‐C levels in the serum of young offspring.

Lipolysis targets larger lipid droplets, resulting in reduced size and newly synthesized lipid droplets that are more susceptible to lipotropic internalization (Schott et al. 2019). Maternal obesity stimulation during the reproductive age can have trans‐generational effects (Hagström et al. 2021), and increased body mass index in early life is closely associated with the occurrence of severe liver disease (Hagström et al. 2018). High‐fat diet in mother mice leads to excessive lipid accumulation in liver tissue cells, resulting in enlarged and increased number of lipid droplets in liver cells. With the increase of obesity in pregnant women, the placental ATP level is significantly reduced, mitochondrial function is abnormal, and the metabolic flexibility of cells is abnormal (Mele et al. 2014). DAG diet has far‐reaching potential to improve the mitochondrial boundary blurring, black deposition, ridge structure destruction and other problems in the liver. The DAG diet improves this unfavorable change, and also improves issues such as blurred mitochondrial boundaries, black deposition, and disrupted ridge structures in liver mitochondria, maintaining the normal structure and morphology of mitochondria, ensuring smooth energy metabolism, and providing good conditions and sites for mitochondrial fatty acid oxidation to proceed smoothly. Through DAG diet intervention of mother mice and their young offspring, lipid deposition in the liver of young offspring was reduced, and the mitochondria structure and morphology of high lipid diet were improved. The mice on the DAG diet had healthier levels of energy metabolism.

During the perinatal period, which is a critical window for cognitive and immune system development (Vinther et al. 2023), it is jointly promoted by maternal and infant gut microbiota and their metabolites (Vatanen et al. 2022). Analysis of 1H‐NMR results of maternal mouse serum showed that the DAG diet partially improves the decreased levels of purine and pyrimidine metabolism in mother mice induced by a high‐fat diet.

Feeding mother mice with a DAG diet results in a partial increase in the gene expression levels of Ppar and its downstream Cpt1a in the liver. PPARα is a crucial factor of lipid homeostasis (Weng et al. 2023), and the increased levels of PPARα and Cpt1a indicate a restoration of the liver's fatty acid metabolism capacity (Mishra et al. 2021). Acss2, a key factor in the conversion of acetate to acetyl‐CoA, can selectively regulate gene expression related to acetate availability, and its deficiency can prevent lipid deposition and obesity (Huang et al. 2018). DAG downregulates Acss2 in the liver and adipose tissue of mice, suggesting a reduction in the production of acetyl‐CoA, an important precursor for de novo synthesis of fatty acids, which may be beneficial for improving obesity. The activation of the DAG‐PKC pathway is associated with various vascular abnormalities in the retina, kidneys, and cardiovascular tissues under diabetic and insulin‐resistant conditions (Das Evcimen and King 2007) and phosphorylated PKC can activate PKD. In mother mice fed with a DAG diet, the expression of Pkc is not abnormally elevated, and the expression of Pkd1 is maintained at normal levels, indicating a healthier vascular status in mother mice on a DAG diet. Maternal obesity can be transmitted between the mother and offspring through epigenetic means (Wang et al. 2018), and intervention in the mother can regulate epigenetic marks by inducing the expression of DNA methyltransferase 1 (Dnmt1) and DNA methyltransferase 3α (Dnmt3α) so as to reduce metabolic syndrome‐like manifestations in the offspring (Izquierdo et al. 2021). Furthermore, the normalization of the expression of Miz‐1, Sim1, and Mmp9—genes previously associated with differential methylation in obesity—in the liver and adipose tissue of mother mice on a DAG diet is crucial for protecting young offspring from the effects of maternal high‐fat diet. It should be emphasized that this study measured mRNA expression rather than DNA methylation; thus, whether DAG intervention directly modulates epigenetic marks requires further investigation. The increased expression levels of Ppar and Cpt1a in the liver and adipose tissue of young offspring indicate that the DAG diet facilitates the smooth progression of fatty acid oxidation pathways in young offspring. The increased expression of Pkc in young offspring of mothers on a high‐fat diet leads to increased vascular tension, which is detrimental to vascular health (Zheng et al. 2023). The DAG diet does not cause abnormal activation of the DAG‐PKC signaling pathway or abnormal expression of Miz‐1, Sim1, and Mmp9.

DNA methylation patterns are associated with gut bacterial populations, mainly in gene promoter regions associated with lipid metabolism and obesity (Kumar et al. 2014), and epigenetic inheritance may be determined by microorganisms and their metabolites (Manterola et al. 2021). Intestinal flora is a key factor in the regulation of obesity (Michels et al. 2022; Vieira‐Silva et al. 2020), and changes in its abundance are closely related to the development of metabolic syndrome such as obesity (Duan et al. 2021; Mayneris‐Perxachs et al. 2021). The obesity phenotype of mice can be improved by adjusting the ratio of *Firmicutes* to *Bacteroidetes*, which is conducive to reducing the weight and lipid accumulation of mice (Chang et al. 2015). The DAG diet of both mother and offspring improved the F/B ratio of fecal intestinal flora in offspring to a certain extent, making it close to the normal group. These results indicated that DAG could regulate the intestinal flora, which was accompanied by changes in the phenotype and metabolic status of mice. An important feature of intestinal microbial disorders in obese patients is the decreased bacterial abundance of SCFAs in the intestinal microbiota, which leads to reduced SCFAs levels and intestinal inflammation (Perez‐Reytor et al. 2021). SCFAs have superior performance in treating obesity and repairing intestinal function (Mei et al. 2024). Butyrate can enhance CPT1A activity, promote fatty acid oxidation and iTreg differentiation (Hao et al. 2021). Administration of sodium butyrate can improve the intestinal barrier while modifying HFD‐induced intestinal microbiota disturbance (Fang et al. 2019). The role of SCFAs is closely related to gut microbes. Some types of microorganisms can play specific roles (De La Cuesta‐Zuluaga et al. 2018). Compared with high‐fat diet, the DAG diet appeared to improve the ratio of *Firmicutes* to *Bacteroidetes* in female microflora and improved the abnormal decrease of *Akkermansia* and *Alloprevotella* relative abundance and the abnormal increase of *Helicobacter* and *Dubosiella* relative abundance caused by high‐fat diet. It improved the abnormal level of SCFAs in high‐fat diet and actively promoted the stable maintenance of SCFAs metabolism and the dynamic balance of intestinal flora in female mice.

During the feeding period of adult offspring, the oN‐ND group had no abnormal behavior, maintained good eating behavior, flexible behavior, and an active mental state, and steadily increased body weight. The growth rate of the oH‐HFD composition was significantly higher than that of the oN‐ND group. Compared with oN‐HFD, the percentage of groin white adipose tissue weight in the oH‐HFD group was significantly increased (*p* < 0.05), but the change was not significant in the oD‐HFD group. The DAG diet of female mice protected their adult offspring from the risk of abnormal accumulation of adipose tissue. Serum TG levels were lower in the oD‐DAG group than in the oH‐DAG group, and serum cholesterol levels were lower in the oD‐DAG group than in the oH‐DAG group. These results indicate that DAG can improve the lipid metabolism of mice fed the same diet with the same proportion of fat content. High levels of triglycerides and cholesterol in the blood can cause hyperlipidemia (Santos‐Baez and Ginsberg 2020), and the development of hyperlipidemia is associated with cardiovascular diseases and pancreatitis (Simha 2020). The increased risk of hyperTGemia in children (Sunil and Ashraf 2022) makes early intervention particularly important. The improvement of TG and T‐CHO in the serum of adult offspring by maternal DAG diet indicates that DAG can prevent the occurrence and development of hyperlipidemia and related risk diseases in adult offspring to a certain extent. However, similar to the results in female mice, the DAG diet did not improve serum HDL‐C levels in adult offspring.

Young and adult offspring show differences in their exposure to the effects of maternal diet. Although the serum lipid level (TG (0.46 ± 0.02) and T‐CHO (2.32 ± 0.25) levels in oDAG group were significantly lower than those in oHFD group, *p* < 0.05) and gene expression (ACSS2 gene expression level in eWAT in oDAG group was significantly lower than that in oHFD group, the infant progeny from different dietary types were significantly lower than those in OHFD group. *p* < 0.01), but body weight gain rate (%) (oDAG group (88.56 ± 3.07) versus oHFD group (91.27 ± 3.27), *p* > 0.05) and body fat content (iWAT: oDAG group (0.53 ± 0.05) versus oHFD group (0.56 ± 0.03), *p* > 0.05; eWAT: There was no significant change in oDAG group (0.39 ± 0.06) versus oHFD group (0.51 ± 0.06), *p* > 0.05). Adult offspring of mothers with different dietary types exhibit different phenotypes when they receive the same diet after weaning, and a healthy maternal diet can make their adult offspring exhibit greater resistance when faced with a high‐fat diet (iWAT: oN‐HFD group (1.05 ± 0.34) vs. oH‐HFD group (1.76 ± 0.24), *p* < 0.05). Although some adult offspring were challenged by their mother's high‐fat diet early in life, the resumption of a healthy diet during the growth phase from weaning to adulthood can help mice maintain a relatively healthy body phenotype into adulthood (iWAT: oH‐ND group (0.41 ± 0.03) vs. oH‐HFD group (1.76 ± 0.24), *p* < 0.05). DAG diet could help the phenotypic recovery of adult progeny (iWAT: oH‐DAG group (0.62 ± 0.11) vs. oH‐HFD group (1.76 ± 0.24), *p* < 0.05; eWAT: oH‐DAG group (1.55 ± 0.31) vs. oH‐HFD group (3.68 ± 0.65), *p* < 0.05; T‐CHO: oH‐DAG group (3.13 ± 0.39) vs. oH‐HFD group (4.98 ± 0.28), *p* < 0.05) promoted intestinal flora homeostasia in adult progeny. The adverse effects of maternal obesity on young offspring can be improved by diet recovery to some extent, and the effects of diet after weaning in their adult years are greater than the effects of maternal diet.

There is a correlation between the effects of DAG on mother mice and their offspring. On the one hand, diglycerol may create more favorable early development conditions for young offspring through the homeostasis of the intrauterine environment; on the other hand, diglycerol appeared to exert a partial protective effect in adult progenies when they face the challenge of a high‐fat diet through cytokines, SCFAs, intestinal flora, etc.

After 6 weeks of FCMT, 16S rRNA gene sequencing of fecal samples showed that the intestinal flora of mice transplanted with fecal suspensions from different diets had varying degrees of structural changes in composition. It was found that the ratio of *Firmicutes* to *Bacteroidetes* increased in the rHFD group. At the species and genus level, *Coryneella alloides*, *Muribaculaceae*, *Muribaculum*, and *Prevotella* in the rDAG group increased in abundance, all of which are closely related to SCFAs, especially butyric acid. The increase in the abundance of butyricogenic bacteria is conducive to the improvement of glycolipid metabolism. Unfortunately, although the use of fecal suspension transplantation technology confirmed that the diglycerol diet could partially improve the intestinal flora disturbances caused by the high‐fat diet, the difference between the three groups could not be reflected in the body weight and body fat of mice (Table 2). Similar studies (Yin et al. 2018) proved that drinking melatonin could alleviate lipid accumulation and intestinal flora disturbance in obese mice, while microbiota transplantation in mice treated with melatonin failed to improve lipid metabolism in mice fed HF diet, making the assumption that the effects of melatonin on intestinal microbiota depend on diet or health status. This phenomenon finds support in human studies, where diet is recognized as a primary driver of gut microbiota composition and function (Segev et al. 2026), and its metabolic effects are often context‐dependent. Furthermore, evidence from human cohorts demonstrates that maternal diet directly shapes the infant's founding gut microbiota (Chu et al. 2016), highlighting the potential for dietary interventions during critical periods to have intergenerational impacts. Thus, the beneficial effects of maternal DAG observed in offspring are likely associated with a diet‐microbiota‐host interplay, where the protective microbial signatures conferred by DAG may require a metabolic challenge (such as the post‐weaning HFD in our study) to fully manifest their anti‐obesity potential.

Several limitations of this study warrant consideration. First, owing to the resource‐intensive nature of multi‐omics profiling (including 1H‐NMR metabolomics, electron microscopy, 16S rRNA sequencing, and SCFA analysis), these assays were restricted to a subset of biologically independent animals (*n* = 3 per group). Although these individuals were randomly selected from larger cohorts (*n* = 6–10 per group) and constitute independent biological replicates, the limited sample size inherently constrains the statistical power and generalizability of these specific findings. Consequently, the observed alterations in mitochondrial morphology, gene expression patterns, serum metabolite profiles, and gut microbiota composition should be interpreted as exploratory and hypothesis‐generating, rather than conclusive. Future investigations with expanded sample sizes are warranted to validate these preliminary observations and to rigorously confirm the robustness of DAG‐mediated metabolic and microbiota alterations in maternal and offspring models. Marked interspecies differences in lipid metabolism—including lipoprotein profiles, bile acid synthesis, and cholesterol homeostasis—limit direct extrapolation of murine findings to human physiology. Rodent cecotrophy, absent in humans, substantially alters gut microbiota composition and short‐chain fatty acid bioavailability, further constraining translational relevance. Future translational studies in human populations or in non‐cecotrophic animal models are required to validate the potential benefits of DAG oil.

## 5 Conclusion

These results showed that maternal DAG diet attenuates selected metabolic effects of maternal HFD or acquired HFD by regulating energy metabolism and lipid metabolism, and is associated with altered microflora and their metabolites, especially intestinal microbial composition. Although DAG can attenuate the adverse effects of maternal obesity on young offspring to a certain extent, the effects of adult diet after weaning are greater than the intrinsic effects of maternal diet.

Our findings provide new insights into the complex role of DAG‐induced changes in maternal metabolism and gut microflora and progeny HFD exposure on the development of progeny obesity in mice, and may inform future preclinical research in rodent models. As a healthy oil, diglyceryl edible oil may help modulate intergenerational metabolic risk in this murine model and potentially attenuate the metabolic burden of obesity.

## Author Contributions

**Qiu Wu:** writing – review and editing, conceptualization. **Chun Li:** investigation, writing – original draft. **Shihao Song:** methodology. **Hanyue Wang:** software. **Rentang Zhao:** data curation. **Luyan Sun:** validation. **Bingqing Shang:** visualization. **Yue Geng:** funding acquisition, project administration, resources, supervision, formal analysis.

## Funding

The study was supported by the Natural Science Foundation of Shandong Province, China [grant number ZR2023QC105] and 2024 Liaocheng Science and Technology help small and medium‐sized enterprises climbing plan project [grant number 2024PDJH42].

## Conflicts of Interest

The authors declare no conflicts of interest.

## Supporting information


**Table S1:** Differential metabolite information table between mND and mHFD groups (*n* = 3).
**Table S2:** Differential metabolite information table between mHFD and mDAG groups (*n* = 3).

## Data Availability

The raw 16S rRNA sequencing data reported in this paper have been deposited in the NCBI Sequence Read Archive (SRA) under BioProject ID PRJNA1520857. The metabolomics data have been deposited in the MetaboLights database under accession number MTBLS15510. The supplementary data (Tables S1 and S2) are provided in the online Supporting Information accompanying this article.
