# Supplementary material for: Maternal Diglyceride Diet Attenuates Selected Metabolic Effects of Maternal High‐Fat Feeding in Offspring via Energy Metabolism and Microbiota Modulation
Source: Food Sci Nutr. 2026 Oct 13;14(10):e72427. doi: 10.1002/fsn3.72427 (PMC13659399; doi:10.1002/fsn3.72427)
Supplement: Supplementary file 1 [file FSN3-14-e72427-s001.docx]

Table S1 Differential metabolite information table between mND and mHFD groups (n=3)

| Name | VIP | Matching shifts | Trend |
| --- | --- | --- | --- |
| 5-methyluridine | 1.67028 | 4.3428 | ↓ |
| Cytidine | 1.59459 | 4.3088 | ↓ |
| Inosine | 1.8955 | 4.4348 | ↓ |
| Guanosine | 1.79704 | 4.3948 | ↓ |
| Thymidine | 1.95753 | 4.4548 | ↓ |
| Uridine | 1.64899 | 4.3348 | ↓ |
| Xanthosine | 1.79314 | 4.3968 | ↓ |
| Adenosine | 1.9116 | 4.4388 | ↓ |
| 2'-Deoxyuridine | 1.98345 | 4.4588 | ↓ |
| NADH | 1.76771 | 4.3868 | ↓ |
| cyclic AMP | 1.98369 | 4.4608 | ↓ |
| FAD | 2.26495 | 4.5189 | ↓ |
| FMN | 2.12286 | 4.0167 | ↑ |
| AMP | 2.32796 | 4.5269 | ↓ |
| ATP | 1.90448 | 4.4308 | ↓ |
| ADP | 2.33135 | 4.5289 | ↓ |
| CDP | 1.8356 | 4.4148 | ↓ |
| GDP | 1.63733 | 4.3288 | ↓ |
| UDP | 1.79383 | 4.4008 | ↓ |
| UMP | 1.65011 | 4.3388 | ↓ |
| 1,6-anhydro-beta-d-glucose | 1.50161 | 3.6627 | ↑ |
| D- (-)-Fructose | 1.72431 | 4.0287 | ↓ |
| D- (+)-Galactose | 2.61335 | 4.5669 | ↓ |
| D- (+)-Xylose | 1.68341 | 4.3468 | ↓ |
| L-Gulonolactone | 2.57341 | 4.5489 | ↓ |
| D-Ribose 5-phosphate | 1.57772 | 4.3048 | ↓ |
| D-Galacturonic acid | 1.80898 | 4.4048 | ↓ |
| lactulose | 2.60118 | 4.5509 | ↓ |
| 3-Fucosyllactose | 1.94272 | 4.4508 | ↓ |
| Raffinose | 2.97529 | 4.0327 | ↑ |
| 1-Kestose | 2.62978 | 4.0347 | ↑ |
| i-Erythritol | 1.50161 | 3.6627 | ↑ |
| Lactose | 1.93277 | 4.4448 | ↓ |
| L-Arabitol | 1.50161 | 3.6627 | ↑ |
| scyllo-Inositol | 1.54762 | 3.3246 | ↑ |
| D-Xylulose | 1.76758 | 4.3828 | ↓ |
| Polygalacturonic acid | 1.78327 | 4.3908 | ↓ |
| L-Canavanine | 1.56707 | 4.0467 | ↑ |
| L-Citrulline | 1.71229 | 3.1285 | ↑ |
| gly-pro | 1.50475 | 4.2788 | ↓ |
| L-Anserine | 2.12259 | 4.4928 | ↓ |
| L-Carnosine | 1.97194 | 4.4568 | ↓ |
| Ala-Ala | 1.59293 | 4.0087 | ↓ |
| N-Acetylhistidine | 1.94147 | 4.4488 | ↓ |
| 2-Hydroxyisocaproic acid | 2.97529 | 4.0327 | ↑ |
| N-Acetylneuraminic acid | 1.97613 | 4.0127 | ↑ |
| O-Acetyl-L-serine | 2.10226 | 4.4868 | ↓ |
| N-acetyl-L-aspartic acid | 1.77591 | 4.3888 | ↓ |
| Argininosuccinic acid | 1.50678 | 4.2768 | ↓ |
| 1-amino-2-propanol | 2.96386 | 1.2401 | ↑ |
| 2-Amino-1-phenylethanol | 1.54762 | 3.3246 | ↑ |
| Cyclohexylamine | 1.5877 | 3.1305 | ↑ |
| 2-hydroxyoctanoic acid | 2.5383 | 1.2661 | ↓ |
| Hexanoic acid | 2.5383 | 1.2661 | ↓ |
| 5-(Hydroxymethyl) uracil | 1.77222 | 4.3848 | ↓ |
| Creatinine | 2.00037 | 4.0488 | ↑ |
| N-cyclohexylformamide | 1.94167 | 1.2681 | ↓ |
| Itaconic acid | 1.85933 | 3.1366 | ↑ |
| Shikimic acid | 1.79444 | 4.3928 | ↓ |
| Isethionic acid | 2.08922 | 3.1345 | ↑ |
| alpha-D-Galactose 1-phosphate | 1.60272 | 3.9927 | ↓ |
| D-pinitol | 1.63484 | 3.9947 | ↓ |
| D-Ribulose 5-phosphate | 1.55099 | 4.3008 | ↓ |
| D-Saccharate | 1.57694 | 4.0528 | ↑ |
| Inosine 5'-monophosphate | 2.30695 | 4.5249 | ↓ |
| Kanamycin | 2.12286 | 4.0167 | ↑ |
| L-Ascorbate | 2.22768 | 4.5129 | ↓ |
| Pyridoxal 5'-phosphate | 2.10672 | 5.0910 | ↓ |
| Pyridoxamine | 1.52759 | 4.2928 | ↓ |

Table S2 Differential metabolite information table between mHFD and mDAG groups (n=3)

| Name | VIP | Matching shifts | Trend |
| --- | --- | --- | --- |
| D-Ribulose 5-phosphate | 1.70452 | 4.04274 | ↑ |
| NADH | 1.97652 | 4.53085 | ↑ |
| Inosine 5'-monophosphate | 1.50629 | 4.37682 | ↑ |
| UDP | 1.58089 | 4.41483 | ↑ |
| UMP | 1.84636 | 4.00273 | ↑ |
| GDP | 2.31427 | 4.59286 | ↓ |
| 2'-Deoxyinosine | 1.80267 | 3.79669 | ↑ |
| CDP | 1.58607 | 4.41282 | ↑ |
| FAD | 1.90071 | 4.51885 | ↑ |
| FMN | 2.2751 | 4.02274 | ↑ |
| cyclic AMP | 1.71149 | 4.46084 | ↑ |
| ATP | 1.65135 | 4.43283 | ↑ |
| ADP | 2.00453 | 4.54285 | ↑ |
| Inosine | 1.68698 | 4.44883 | ↑ |
| 2'-Deoxyguanosine | 1.86858 | 4.60487 | ↓ |
| Xanthosine | 1.54407 | 4.40482 | ↑ |
| Uridine | 1.80267 | 3.79669 | ↑ |
| Adenosine | 1.66855 | 4.43483 | ↑ |
| 2'-Deoxyuridine | 1.69622 | 4.45683 | ↑ |
| Guanosine | 1.57986 | 4.40882 | ↑ |
| Dihydrouracil | 1.57647 | 3.43661 | ↑ |
| Cytidine | 1.6516 | 3.79869 | ↑ |
| Thymidine | 1.74407 | 4.47084 | ↑ |
| 3,4-Dihydroxy-L-phenylalanine | 1.54446 | 3.14255 | ↑ |
| 5-Hydroxy-L-tryptophan | 2.63618 | 4.02074 | ↑ |
| L-Canavanine | 1.59945 | 4.03674 | ↑ |
| L-Proline | 1.78102 | 3.36259 | ↑ |
| L-Valine | 1.6553 | 3.60465 | ↑ |
| L-Tryptophan | 2.36839 | 4.03474 | ↓ |
| L-Isoleucine | 4.124 | 1.25013 | ↑ |
| L-Kynurenine | 1.5651 | 3.70467 | ↑ |
| 3-Ureidopropionic acid | 1.50415 | 3.29458 | ↑ |
| N-Acetylhistidine | 1.69712 | 4.45083 | ↑ |
| 2-Hydroxyisocaproic acid | 1.5734 | 4.02674 | ↑ |
| n-acetylglycine | 1.66344 | 3.74068 | ↑ |
| N-Acetylneuraminic acid | 1.68352 | 4.02474 | ↑ |
| O-Acetyl-L-serine | 1.83966 | 4.49884 | ↑ |
| Kanamycin | 1.62502 | 3.36059 | ↑ |
| N6-Acetyl-L-lysine | 1.73572 | 3.16655 | ↑ |
| Ala-Ala | 2.04934 | 4.01474 | ↓ |
| D- (-)-Fructose | 3.05777 | 4.03274 | ↓ |
| D- (+)-Xylose | 2.30244 | 4.56286 | ↑ |
| Lactulose | 2.71706 | 4.55486 | ↑ |
| D- (+)-Maltose | 1.66127 | 3.41461 | ↑ |
| 1-Kestose | 1.6516 | 3.79869 | ↑ |
| Raffinose | 2.24525 | 4.55286 | ↑ |
| 3-Fucosyllactose | 1.68596 | 4.44683 | ↑ |
| meso-Erythritol | 1.69007 | 3.62865 | ↑ |
| i-Erythritol | 1.54251 | 3.70667 | ↑ |
| D-Mannose | 1.70309 | 3.3646 | ↑ |
| D-Ribose 5-phosphate | 2.02005 | 4.05075 | ↓ |
| D-Galacturonic acid | 1.55134 | 4.40082 | ↑ |
| Dihydroxyacetone | 1.58309 | 4.41082 | ↑ |
| D-Xylulose | 1.51986 | 4.38082 | ↑ |
| D-Sorbitol | 1.72174 | 3.62665 | ↑ |
| D-Fructose-6-phosphate | 1.79778 | 3.65266 | ↑ |
| alpha-D-Glucose 1,6-bisphosphate | 1.91639 | 4.01674 | ↓ |
| Xylitol | 1.62041 | 3.63465 | ↑ |
| 5-(Hydroxymethyl)uracil | 1.51216 | 4.38482 | ↑ |
| Adonitol | 1.51988 | 3.69267 | ↑ |
| Methanol | 1.70636 | 3.34059 | ↑ |
| D-pinitol | 1.86306 | 3.33059 | ↑ |
| D-Mannitol | 1.52867 | 3.79469 | ↑ |
| L-Arabitol | 1.56563 | 3.74268 | ↑ |
| L-(-)-Fucose | 6.668 | 1.24413 | ↓ |
| L-(-)-Threitol | 1.65441 | 3.65466 | ↑ |
| Polygalacturonic acid | 1.57165 | 4.40682 | ↑ |
| scyllo-Inositol | 1.96033 | 3.32859 | ↑ |
| N-Acetyl-D-glucosamine | 1.60037 | 3.8267 | ↑ |
| 1,6-anhydro-beta-d-glucose | 1.54363 | 3.67266 | ↑ |
| Lactose | 1.69796 | 4.45283 | ↑ |
| Hexanoic acid | 1.84843 | 1.26614 | ↑ |
| 2-hydroxyoctanoic acid | 1.67688 | 4.00474 | ↑ |
| 3,4-Dihydroxyphenylacetic acid | 1.62241 | 3.3786 | ↑ |
| 4-Hydroxyphenylacetic acid | 1.53394 | 3.43461 | ↑ |
| 3-Methyl-2-oxobutinoic acid | 2.98312 | 1.1141 | ↓ |
| Homovanillic acid | 1.57647 | 3.43661 | ↑ |
| 3-Methyl-2-oxopentanoic acid | 2.01505 | 1.0961 | ↓ |
| Itaconic acid | 1.54446 | 3.14255 | ↑ |
| Shikimic acid | 1.54308 | 4.39282 | ↑ |
| 4-Acetamidobutyric acid | 1.50187 | 3.16455 | ↑ |
| 4-Guanidinobutyric acid | 1.93187 | 3.18656 | ↑ |
| Hypotaurine | 1.70439 | 3.34459 | ↑ |
| Isethionic acid | 1.54446 | 3.14255 | ↑ |
| D-Glucono-1,5-lactone | 1.66344 | 3.74068 | ↑ |
| Cysteamine | 1.80456 | 3.3906 | ↑ |
| N-cyclohexylformamide | 1.84843 | 1.26614 | ↑ |
| Creatinine | 2.58494 | 4.03874 | ↑ |
| Cyclohexylamine | 1.74098 | 1.15411 | ↓ |
| 2-Amino-1-phenylethanol | 1.75456 | 3.31859 | ↑ |
| triethanolamine | 1.56258 | 3.3966 | ↑ |
| L-Ascorbate | 1.8836 | 4.51285 | ↑ |
| Pantothenate | 1.57647 | 3.43661 | ↑ |
| D-Glyceraldehyde | 1.51988 | 3.69267 | ↑ |
| vanillin | 1.56316 | 3.8287 | ↑ |
